# Interactions with soil fungi alter density dependence and neighborhood effects in a locally abundant dipterocarp species

**DOI:** 10.1002/ece3.8478

**Published:** 2022-01-24

**Authors:** R. Max Segnitz, Sabrina E. Russo, Kabir G. Peay

**Affiliations:** ^1^ Department of Medicine University of Washington Seattle Washington USA; ^2^ School of Biological Sciences University of Nebraska Lincoln Nebraska USA; ^3^ Center for Plant Science Innovation University of Nebraska Lincoln Nebraska USA; ^4^ Department of Biology Stanford University Stanford California USA; ^5^ Woods Institute for the Environment Stanford California USA

**Keywords:** Borneo, coexistence, ectomycorrhiza, feedbacks, fungi, Janzen–Connell, Lambir Hills, natural enemies, oomycete, pathogen, population

## Abstract

Seedling recruitment can be strongly affected by the composition of nearby plant species. At the neighborhood scale (on the order of tens of meters), adult conspecifics can modify soil chemistry and the presence of host microbes (pathogens and mutualists) across their combined canopy area or rooting zones. At local or small spatial scales (on the order of one to few meters), conspecific seed or seedling density can influence the strength of intraspecific light and resource competition and also modify the density‐dependent spread of natural enemies such as pathogens or invertebrate predators. Intrinsic correlation between proximity to adult conspecifics (i.e., recruitment neighborhood) and local seedling density, arising from dispersal, makes it difficult to separate the independent and interactive factors that contribute to recruitment success. Here, we present a field experiment in which we manipulated both the recruitment neighborhood and seedling density to explore how they interact to influence the growth and survival of *Dryobalanops aromatica*, a dominant ectomycorrhizal tree species in a Bornean tropical rainforest. First, we found that both local seedling density and recruitment neighborhood had effects on performance of *D*. *aromatica* seedlings, though the nature of these impacts varied between growth and survival. Second, we did not find strong evidence that the effect of density on seedling survival is dependent on the presence of conspecific adult trees. However, accumulation of mutualistic fungi beneath conspecifics adults does facilitate establishment of *D*. *aromatica* seedlings. In total, our results suggest that recruitment near adult conspecifics was not associated with a performance cost and may have weakly benefitted recruiting seedlings. Positive effects of conspecifics may be a factor facilitating the regional hyperabundance of this species. Synthesis: Our results provide support for the idea that dominant species in diverse forests may escape the localized recruitment suppression that limits abundance in rarer species.

## Graphical abstract

We present a rare‐field experiment in which we manipulated both the recruitment neighborhood (whether or not seedlings recruited in proximity to adult conspecifics) and seedling density to explore how they interact to influence the growth and survival of a dominant ectomycorrhizal tree species in a Bornean tropical rainforest. Our results provide support for the idea that dominant species in diverse forests may escape the localized recruitment suppression that limits abundance in rarer species and that ectomycorrhizal fungi play an important role in reducing CNDD for some tropical trees.

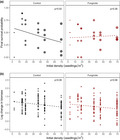

## 1 INTRODUCTION

Factors that influence tree recruitment at early life stages play a critical role in determining the realized diversity and community composition of mature forests (Bagchi et al., 2014; Harms et al., 2000; Hubbell et al., 1999). Because plants are sessile, seed dispersal plays a critical role in determining the quality of the abiotic environment and the types of biotic interactions seedlings experience (Russo & Augspurger, 2004). For example, seeds that disperse short distances must establish in soils conditioned by root and leaf litter input from the parent tree and generally encounter higher density of conspecific seedlings. Local density of conspecific seedlings and proximity to conspecific adults impact both the extent of intraspecific interactions (Levine & Murrell, 2003) and the likelihood of attack from natural enemies (Augspurger, 1983b; Connell, 1971; Janzen, 1970). The cumulative effect of intraspecific competition and natural enemy attack is to generate a negative relationship between conspecific density and seedling mortality during recruitment. While there is debate about how to quantify the strength of conspecific negative density dependence (CNDD) (Chisholm & Fung, 2018; Hulsmann & Hartig, 2018; LaManna et al., 2018), CNDD is thought to be common and play a major role in the recruitment process (Comita et al., 2010; Harms et al., 2000; LaManna et al., 2017; but see Detto et al., 2019). However, not all species experience the same degree of CNDD, and variation in the strength of CNDD can be a good predictor of tropical tree commonness and rarity (Comita et al., 2010). Understanding what controls the strength of CNDD and why it varies between species is thus a priority for understanding tropical forest community structure and diversity maintenance (Stump & Comita, 2018).

One difficulty in understanding CNDD is that the dispersal process results in an intrinsic correlation between local‐scale seedling density and the recruitment neighborhood. Recruitment neighborhood refers to the spatially bounded effects of a parent tree on soil chemistry and microbial communities and also referred to as the ‘distance effect’ in some studies. Correlation between recruitment neighborhood and conspecific seedling density makes it difficult to separate the independent and interactive factors that affect CNDD and contribute to recruitment success (Augspurger, 1983a; Augspurger & Kitajima, 1992). For example, the negative effects of fungal pathogens are increasingly recognized as playing an important role in generating CNDD and controlling tree seedling recruitment (Augspurger & Kelly, 1984; Gilbert et al., 2001; Packer & Clay, 2000). The activity of fungal pathogens is itself density‐dependent (i.e., with greater spread at higher host density; Gilbert, 2002; Liu et al., 2012), while at the same time, plant–soil feedbacks (PSFs) increase the abundance of host‐specific fungal pathogens in soils conditioned by conspecific adult trees (Bagchi et al., 2014; Mangan et al., 2010; Segnitz et al., 2020). Because seedling density is inevitably higher closer to adult conspecifics in the field, along with other factors that might reduce or promote seedling establishment, experimental approaches are necessary to understand the importance of adult conspecifics and host‐specific fungal pathogens in generating CNDD.

Despite the importance of CNDD, field experiments that manipulate seedling density, seedling neighborhood, and the presence of fungal pathogens in order to parse the effects of these drivers of CNDD on seedling recruitment over an extended period of time are rare. Few studies have looked at the effects of conspecific soil conditioning on seedling survival in controlled field manipulations (but see Yamazaki et al., 2008), and a very limited number of studies have sought to experimentally disentangle effects of seed/seedling density and dispersal (Augspurger & Kelly, 1984; Takeuchi & Nakashizuka, 2007). We know of only one published study that focused on the intersection of density‐dependent mortality and PSF in a neighborhood framework, which found that neighborhood and density effects were common in a temperate forest (Yamazaki et al., 2008). Further, most work on PSF has examined recruitment in terms of the seed to seedling transition over relatively short timescales, and none specifically manipulated the presence of soil fungi in conjunction with manipulation of field density and neighborhood. As a result, we still know too little about how seedling density, seedling neighborhoods, and the presence of soil fungi interactively impact the survival of established seedlings over the first couple years of recruitment.

While it is clear that species vary in their susceptibility to host‐specific pathogens and in the resulting strength of CNDD (Comita et al., 2010), it is not clear what traits cause these differences to emerge. Numerous studies have demonstrated that soil‐borne microbiota may underlie patterns of CNDD or distance dependence through the process of negative PSF (Mangan et al., 2010; Packer & Clay, 2000; Teste et al., 2017; Van der Putten et al., 1993). Specificity in pathogen host range and severity of impact on the host can drive feedbacks and neighborhood effects on seedling mortality, with the composition of the local tree community strongly affecting seedling survival at various scales (Bagchi et al., 2010; Hantsch et al., 2014; Webb et al., 2006). Still, interactions with soil biota are complex, and feedback dynamics may be context‐dependent and largely contingent on the natural history of the species involved (Corrales et al., 2016). While proximity to adult conspecifics often suppresses the growth or survival of seedlings due to accumulated pathogens or competitive interactions (Bever et al., 2015; Mangan et al., 2010; Metz et al., 2010; Seiwa et al., 2008), they can also provide a growth benefit due to shared microbial symbionts such as mycorrhizal fungi (Bennett et al., 2017; Segnitz et al., 2020). The positive effects of mycorrhizal fungi potentially can offset the negative effects of density dependence (Bachelot et al., 2017; Liang et al., 2015). Several studies of ectomycorrhizal (EM) tree species across tropical regions suggest that the survival and/or growth of EM recruits can be enhanced by the local abundance of EM adults (McGuire, 2007; Newbery et al., 2000), or physical proximity to adult conspecifics of other adult EM trees (Brearley et al., 2017; Onguene & Kuyper, 2014).

To parse the complex drivers of CNDD and its effects on seedling recruitment, we used a manipulative field experiment to explore interacting effects of local‐scale seedling density, recruitment neighborhood (either adult conspecifics or heterospecifics), and the presence of soil fungi on recruitment of *Dryobalanops aromatica*, a dominant ectomycorrhizal tree in Bornean tropical rainforests. We planted and tracked 4‐year growth and survival of almost 700 seedlings of *D*. *aromatica* in the field at a wide range of densities, in both conspecific or heterospecific adult tree neighborhoods and with or without a fungicide treatment. We predicted that growth and survival would (a) decline more rapidly with seedling density in conspecific neighborhoods due to higher fungal disease risk, but (b) would on average be higher near conspecifics, owing to increased inoculation by EM fungi. If soil fungi are at least partly responsible for the density and neighborhood effects on seedling growth and survival, then the interactive effects between density and neighborhood would be removed or reduced by the application of fungicide.

## 2 METHODS

### 2.1 Study site and species

We conducted this work at Lambir Hills National Park (LHNP) in Sarawak, Malaysia (4°20′N, 113°50′E), a 7800‐ha protected area in northwest Borneo classified as tropical mixed dipterocarp forest. Over 1200 species occur in a 52‐ha long‐term Forest Dynamics Plot (FDP) at LHNP, with dominance by species of the Dipterocarpaceae, which account for approximately 16% of stems and 42% of basal area (Lee et al., 2002).

In 2014, following a general flowering event at LHNP, we established 48 experimental field plots in which we manipulated density and distance to adult conspecifics of recruiting seedlings in a single seedling species, *Dryobalanops aromatica* Gaertn.f. (Dipterocarpaceae). Although the Dipterocarpaceae are considered to be strongly ectomycorrhizal (Brundrett, 2009), we also confirmed the ectomycorrhizal (EM) status of *D*. *aromatica* in a separate greenhouse study (Segnitz et al., 2020). *D*. *aromatica* is a locally abundant species, which occurs at high density and can be monodominant in certain locations of peninsular Malaysia (Hart et al., 1989; Nik Norafida et al., 2018). *D*. *aromatica* does not form monotypic stands at LHNP, though it is the most abundant species in the 52 ha FDP both by number of stems as well as by basal area, accounting for approximately 2.5% of all stems >1 cm diameter at breast height and 6.7% of total basal area (Itoh et al., 2003). Our experiment was established outside of, but in an area adjacent to the FDP, making ecological data about this species from the FDP directly relevant to our study.

### 2.2 Experimental design

In June and July of 2014, we collected seeds from 18 individuals of *D*. *aromatica*. Seeds were collected prior to or in very early stages of germination. We did not collect seeds if the emerged radicals had touched the litter on the forest floor. Seeds were not surface sterilized due to speed of germination (*D*. *aromatica* seeds have no dormancy and germinate shortly after or immediately upon dropping) and concern that this would kill the seedlings. However, we washed seeds thoroughly to remove any soil and then rinsed them in sterile water before germination in individual small potting bags of washed river sand. Seedlings were allowed to grow in sandbags until transplant to the field.

In September and October, 2014 (9/18/14–10/4/14), we transplanted seedlings into the field, establishing experimental plots at six different density treatments, with 4, 9, 12, 16, 20, or 25 seedlings per 40 cm × 40 cm plot. We established a boundary around each 40 cm × 40 cm planted area such that the seedlings had no naturally present conspecific neighbors nearer than the outplanted seedlings, thus generating seedling densities of 3, 14, 19, 37, 46, and 70 seedlings/m^2^. Plots were distributed among 12 sites, designated either as ‘conspecific neighborhood’ sites centered around an adult *D*. *aromatica*, or ‘heterospecific neighborhood’ sites, which we defined as centered around an adult tree not in the Dipterocarpaceae and with no *D*. *aromatica* >20 cm DBH within 15 m. At all sites, we counted and mapped any *D*. *aromatica* >20 cm DBH to ensure that none were present at all Away sites. At each site, we established four seedling plots, each 5 m from the central tree. Within each site, these four plots consisted of two different density levels, with one plot of each density level receiving fortnightly application of the fungicide Captan WP at a rate of 2.5 g/m^2^ (in aqueous solution) following Segnitz et al. (2020) and Liu et al. (2012). Nonfungicide plots were treated with an equivalent volume of water on the same schedule. Captan is a metalaxyl‐based broad‐spectrum fungicide acting on a wide range of fungi including both Oomycete and fungal pathogens (Cohen & Coffey, 1986; Martınez‐Toledo et al., 1988) and reduced root colonization by EM fungi in a previous dipterocarp experiment (Segnitz et al., 2020). We established two replicate plots of each *neighborhood* × *density* × *fungicide* treatment combination, comprising a total of 48 plots containing 688 seedlings (6 density levels × 2 fungicide groups × 2 home/away groups × 2 replicates = 48 plots). *D*. *aromatica* shows preference for soils with higher sand content at LHNP (Itoh et al., 2003), and all plots were established in a single, sandy soil type, and statistical models included a random effect to account for unmeasured microhabitat variability.

Seedlings were censused regularly for mortality every 2 weeks from the start of the experiment in September 2014 until April 2017 (~2.5 years), after which they were censused twice, in June 2017 and July 2018. Fungicide application was discontinued along with regular censusing beginning in April 2017 due to logistical constraints.

At the start and end of the experiment, as well as several time points throughout, we measured stem diameter, height, leaf number, and estimated total leaf damage for each seedling in the experiment (growth censuses in September 2014, May 2015, September 2016, June 2017, July 2018). While estimates of leaf damage did not distinguish between insect and pathogen damage, we recorded instances in which seedling mortality occurred following stem clipping.

### 2.3 Data analysis

We took several approaches to examining the performance of seedlings in the plot network over the roughly 4‐year course of the experiment. Primary analysis presented within this paper focuses on final growth and survival data through July 2018; however, recognizing that a substantial change occurred with the cessation of fungicide application after June 2017, we also present parallel analyses using data from the final census prior to cessation. These analyses indicate no notable differences from the results presented here and are included in the supplemental materials (Tables S1, S2 and S5). All analyses were conducted in R (R Core Development Team, 2018).

#### 2.3.1 Seedling survival through time

To estimate the survival of individual seedlings through time, we used survival analysis to fit Kaplan–Meier (KM) curves to repeated census data for all 688 seedlings in the experiment. We fit KM curves to all treatment groups (starting density, neighborhood, fungicide application) and used Mantel–Cox log‐rank tests to determine differences in the survival distributions across groups. While Kaplan–Meier estimation does provide insight into temporal changes in seedling survival, it is only appropriate for univariate analysis and cannot incorporate potential interactions among treatments.

#### 2.3.2 Survival outcomes in the plot network

To assess seedling mortality, we calculated the proportion of seedlings in each plot surviving to July 2018. As its distribution is bounded by 0 and 1, proportional survival was analyzed using a generalized linear mixed model (GLMM) following a binomial error distribution, weighted by the number of seedlings in each plot. This approach effectively models seedlings in each plot as success/failure trials in which the outcome is either survival (success = 1) or mortality (failure = 0). This modeling approach inherently reduces the statistical weighting of the lowest density plots, which may be more susceptible to dramatic reduction in percent survival driven by chance events. Our model included the experimental treatments of starting density, neighborhood (conspecific vs. heterospecific), and fungicide application as independent variables and site as a random effect. As our experimental treatments were intentionally designed to test specific hypotheses, we retained all treatment main effects and all 2‐way and 3‐way interactions in our base statistical model. We tested whether to include nonlinear effects of initial density by comparing the base model to models including second‐ and third‐order polynomial terms for the effect of density and found that these terms reduced model fit as determined by AIC. As overdispersion was initially indicated by testing the distribution of Pearson residuals against the *χ* ^2^ distribution following (Bolker et al., 2009), we fit final models by beta‐binomial GLMM as advocated by Harrison (2015).

Beta‐binomial GLMMs were fit using the package ‘glmmADMB’ (Fournier et al., 2012). Inferential tests were conducted using the ‘car’ package (Fox & Weisberg, 2011). To explore higher order interactions, we calculated estimates and variance of the within‐interaction slopes using ‘simple slopes’ analysis (Aiken & West, 1991; Preacher et al., 2006), implemented in the package ‘interactions’ (Long, 2019). Calculation of simple slopes is a form of interaction test for moderated multiple regression models that tests whether the slope of a response variable differs from zero across another categorical (or continuous) predictor (e.g., the relationship between seedling survival and density across fungicide treatments), similar to an analysis of covariance (ANCOVA) test for slope differences across categorical factors.

### 2.4 Seedling growth response

#### 2.4.1 Biomass estimation

To analyze the accumulation of biomass by seedlings throughout the experiment, we first used allometric models and seedling measurements made in the field to estimate the biomass of all seedlings in the experiment at several time points. Allometric models were used in order to be able to estimate biomass at multiple time points and because no destructive harvest of the experiment had been undertaken as of December 2018. The biomass prediction model was trained on data from a previous study at the same site in which 147 *Dryobalanops* seedlings were grown individually in the shadehouse for ~2 years before a destructive harvest and accurate measurements of dry weight (Segnitz et al., 2020). The biomass prediction model was fit using average stem diameter, total stem length, and leaf number as independent variables and explained approximately 92% of variance in seedling total dry weight in the training dataset (Table S3). More details of biomass estimation modeling can be found in the supplement.

#### 2.4.2 Seedling growth response

To assess growth responses among treatment groups, we used a linear mixed effect model (LMM) on the log change in biomass of each seedling over the course of the experiment (log(final biomass) − log(initial biomass). We included a random effect of site to account for unmeasured microhabitat variation and specified a plot‐level random effect within site to address nonindependence of seedlings within a single plot. Our model included all treatments (starting density, neighborhood (conspecific vs. heterospecific), and fungicide application) as independent variables. Again, we retained all main effect treatments and all 2‐way and 3‐way interactions in the model and performed no model selection on model terms, as these treatment interactions were intentionally designed to test specific hypotheses. We visually inspected the model residuals for normality and evidence of heteroskedasticity, which we did not find. LMMs were fitted using the R package ‘lme4’ (Bates et al., 2014) and inferential tests were conducted using the ‘car’ package (Fox & Weisberg, 2011).

Pseudo *R* ^2^ values were calculated following (Nakagawa et al., 2017) as implemented in the R packages ‘performance’ (Lüdecke et al., 2021) and ‘MuMIn’ (Bartoń, 2020) for beta‐binomial generalized linear mixed effects models and linear mixed effects models, respectively.

## 3 RESULTS

### 3.1 Survival through time

**FIGURE 1:**
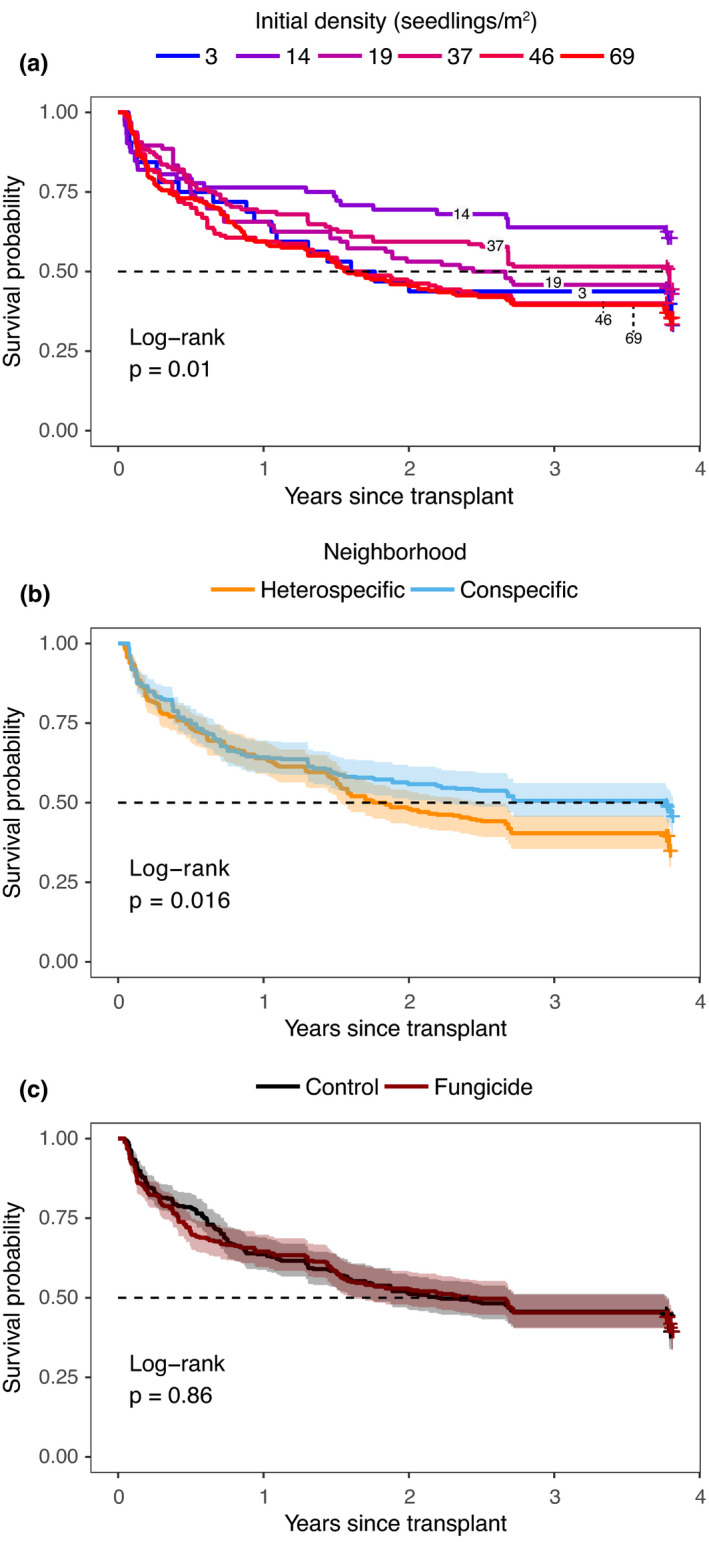
Kaplan–Meier (KM) estimated survivorship curves across treatment groups over the course of the experiment. KM curves are a univariate analysis and do not reflect interactions among main treatments. Nonparametric log‐rank tests indicate significant differences between or among the plotted curves in each panel. The main treatment groups presented are (a) seedling cohorts at six different initial densities, (b) seedling cohorts in conspecific or heterospecific recruitment neighborhoods, and (c) seedling cohorts in control or fungicide groups. Dashed horizontal line indicates 50% seedling mortality

**FIGURE 2:**
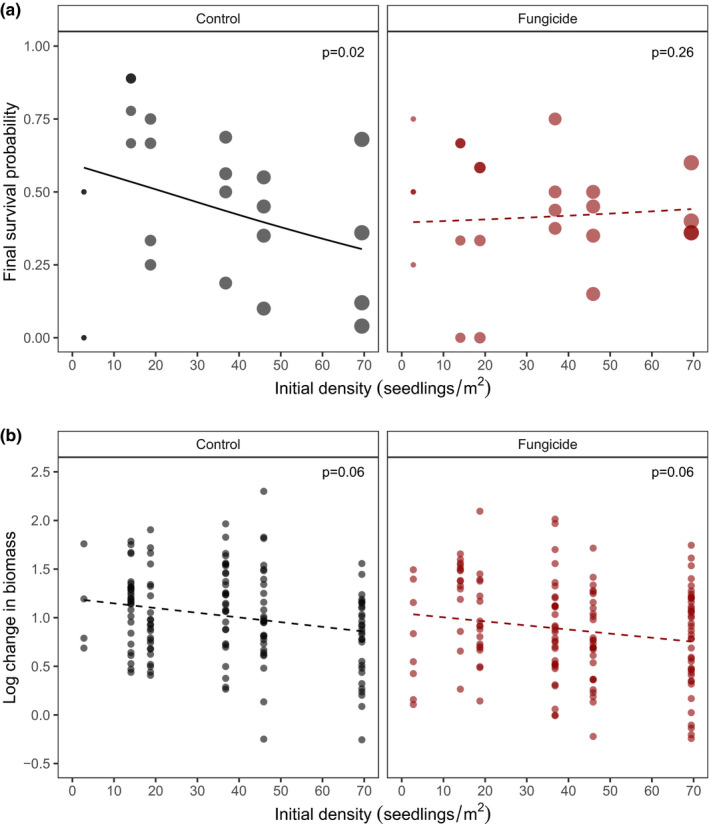
Fungicide moderates the effect of initial seedling density on survival but not growth. (a) Mortality risk increases with initial seedling density in control but not fungicide plots. Trend lines show model prediction estimated from beta‐binomial GLMM model. Point size indicates the number of seedlings per plot (4–25). The *Initial density* × *Fungicide* interaction is significant at *p* < .01, and we present estimated *p* values for individual trends within the interaction derived from simple slopes analysis indicating whether or not the slopes differ significantly from zero. (b) Initial seedling density reduces seedling growth response. The main effect of *Initial density* is marginally significant (*p* = .062). Because the *Initial density* × *Fungicide* interaction was not significant (*p* = .94), no further testing of individual slopes was applied

As expected, survival probability of seedlings decreased steadily through time, with 294 of 688 seedlings (43%) surviving to the end of the experiment (3.8 years after transplant). Survivorship curves showed that both recruitment neighborhood and initial seedling density significantly changed the trajectories of seedling survival through time. Overall survival of seedlings was generally higher in conspecific neighborhoods, although survival curves only began to deviate significantly after around 1.5 years (log‐rank *p* = .016) (Figure 1). Survival curves were also differentiated among density cohorts (log‐rank *p* = .01), with a general trend of lower survival at higher densities (Figure 2). Fungicide had no significant overall effect on survival trends (log‐rank *p* = .86, Figure S2). Of the 395 seedlings that had died by the end of the experiment (57% of initial), 52 (7%) died following stem clipping by an herbivore, and the probability that a seedling death occurred following a stem clipping event was unaffected by our experimental treatments (Table S4).

### 3.2 Survival and growth after 4 years

**FIGURE 3:**
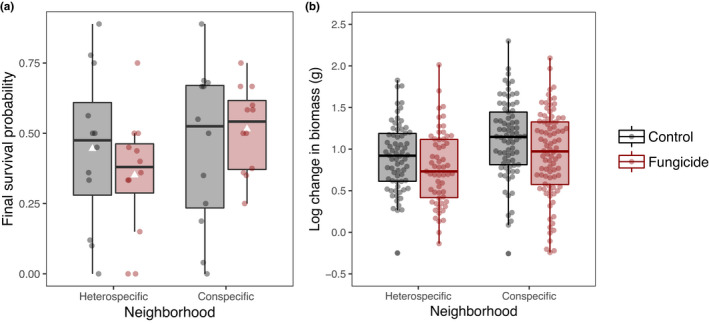
Effects of recruitment neighborhood and fungicide application on seedling mortality and growth response. Boxes indicate the median and interquartile ranges (IQR) of percent survival among plots (a) and growth response among individual seedlings (b), with whiskers extending to data points within 1.5*IQR. White triangles indicate group means. (a) The effect of fungicide effect on seedling mortality depends on recruitment neighborhood. *Fungicide* × *Neighborhood* interaction *p* < .01 in beta‐binomial GLMM. (b) Fungicide consistently reduces growth across neighborhoods. Main effect of *Fungicide* is marginally significant in mixed model ANCOVA (*p* = .051), while effects of *Neighborhood* and the *Neighborhood* × *Fungicide* interaction are not (*p* = .43, *p* > .99, respectively)

Final survival and growth rates of seedlings varied based on planting density, recruitment neighborhood, and fungicide application, with the full model explaining roughly 14% and 32%, respectively, of the variation in these rates (survival *R* ^2^ *conditional* = .14; growth *R* ^2^ *conditional* = .32), whereas for both, fixed effects alone explained roughly 6% of variation (survival *R* ^2^ *marginal* = .06; growth *R* ^2^ *marginal* = .06). The large amount of variance explained by the random effects indicates site‐level variability in seedling performance related to unmeasured biotic factors or microhabitat features, such as light levels or soil nutrients. Multiple interactions also significantly affected survival probability of seedlings, whereas growth response was not sensitive to such interactions (Figures 2 and 3). For survival, the effect of density on seedling survival depended on fungicide application (Density × Fungicide interaction *p* = .008; Figure 2a, Table 1). The ‘simple slopes’ test (similar to an ANCOVA for more complex regression models; see Methods) showed that this interaction resulted from a significant increase in seedling mortality with initial planting density (*p* = .02, Table S5) that was removed by the application of fungicide (*p* = .26, Table S5). Seedling growth response showed a similar trend that was marginally significant, with seedling growth decreasing as the initial seedling density increased (*p* = .06, Figure 2b; Table 2). However, the slope of this relationship was unaffected by fungicide application (Density × Fungicide interaction *p* = .94, Figure 2b; Table 2).

**TABLE 1:** Analysis of deviance table for beta‐binomial generalized linear mixed effects model on percent survival in the plot network. Model includes random effect of site

Fixed effects	*χ* ^2^	df	*p*
Initial density	5.427	1	.020
Neighborhood	0.264	1	.607
Fungicide application	9.450	1	.002
Density × Neighborhood	0.477	1	.490
Density × Fungicide	7.365	1	.007
Neighborhood × Fungicide	4.786	1	.029
Density × Neighborhood × Fungicide	2.359	1	.125
R^2^ *marginal* = .140			
R^2^ *conditional* = .34			

**TABLE 2:** Analysis of covariance table for linear mixed effects model of individual seedling growth responses to experimental treatments. Model includes plot‐level random effect nested within random effect of site

Fixed effects	*χ* ^2^	df	*p*
Initial density	3.473	1	.062
Neighborhood	0.621	1	.431
Fungicide application	3.808	1	.051
Density × Neighborhood	0.213	1	.645
Density × Fungicide	0.006	1	.941
Neighborhood × Fungicide	0.000	1	1.000
Density × Neighborhood × Fungicide	0.000	1	.991
R^2^ *marginal* = .06			
R^2^ *conditional* = .32			

The effect of fungicide on survival also varied significantly between plots in conspecific versus heterospecific neighborhoods (Neighborhood × Fungicide interaction *p* = .029; Table 1). Fungicide had a stronger effect in heterospecific than conspecific neighborhoods, reducing survival in the former but not the latter (Figure 3a). Growth was generally reduced with fungicide treatment (*p* = .05), and this effect was consistent in both conspecific and heterospecific recruitment neighborhoods (Neighborhood × Fungicide p = 1.00). While growth tended to be higher in conspecific neighborhoods, the effect of neighborhood on growth was nonsignificant (*p* = .43; Table 2).

For neither survival nor growth response models was the highest order interaction significant for our statistical models (*survival*: Neighborhood × Fungicide × Density *p* = .12; Table 1; *growth*: Neighborhood × Fungicide × Density, *p* = .99; Table 1). The direction of the 3‐way interaction was consistent with the interpretation that fungi have more positive effects on *D*. *aromatica* survival in conspecific neighborhoods (Figure S1).

## 4 DISCUSSION

That the survival of tree seedlings is locally density‐dependent has been well established. Yet, how contingent these effects are on neighborhood and what are the predominant causal agents are still not well understood. In a 4‐year‐long factorial field experiment, we found that these factors interacted in complex ways to affect growth and survival of the seedlings of a dominant ectomycorrhizal tree species, *D*. *aromatica*, in a Bornean rain forest. Our results indicated that interactions with fungi can have both negative and positive effects on seedling establishment, in that local negative density dependence of survival was mediated by fungi, yet the presence of fungi tended to enhance seeding growth. While our results did not provide strong evidence that being in a conspecific or heterospecific recruitment neighborhood affected the strength of density‐dependent seedling survival in this tree species, seedling survival was higher in conspecific neighborhoods. Our experimental finding is contrary to many observational studies of the effects of neighborhood density of conspecific seedlings and adults on seedling survival in tropical forests, which have often found conspecific negative density‐dependent effects of both seedlings and adults on seedling survival (Bagchi et al., 2014; Comita et al., 2010; Harms et al., 2000; Mangan et al., 2010). The difference between ours and results from these studies may owe to the ability of our experiment, which manipulated seedling density and recruitment neighborhood in a fully crossed design, to separate the independent effects of seedling and adult density, which are confounded in observational studies because in nature seedlings tend to be aggregated near parents, where most seeds land (Condit et al., 2000; Russo & Augspurger, 2004). However, our results are consistent with an experimental study of plant–soil feedbacks in the same forest, which showed that *D*. *aromatica* grown in soil collected from underneath conspecific and closely related species grew faster and had higher colonization rates of ectomycorrhizal fungi than when grown in soil from more distantly related tree species (Segnitz et al., 2020). In heterospecific neighborhoods, disruption of naturally functioning fungal communities through fungicide application decreased seedling survival, whereas application of fungicide did not change survival in conspecific neighborhoods. One explanation for this difference is that ectomycorrhizal fungal inoculum that is most beneficial for *D*. *aromatica* is less abundant in heterospecific neighborhoods, and thus, fungicide application may more effectively eliminate these relationships. While there are alternative explanations—for example, fungicide may have promoted generalist pathogen communities in heterospecific neighborhoods—this interpretation is consistent with our previous studies of ectomycorrhizal inoculum potential (Peay et al., 2012; Segnitz et al., 2020) and the overall higher survival of seedlings in conspecific sites in this study. In the soils of this Bornean rain forest, there is a wide diversity of mutualist, commensal, and pathogenic fungi (Peay et al., 2010, 2015; Sato et al., 2015), and our findings suggest that the effect of these seedling–fungal interactions can vary in intensity and net effect across the forest. Since these interactions depended on seedling density and the recruitment neighborhood, they can generate complex, but predictable, patterns of intraspecific spatial variation in the strength and direction of density‐dependent growth and survival of seedlings. To the extent that similar types of contingent seedling–fungal interactions we observed for *D*. *aromatica* generalize to other species, they may strongly affect diversity and dominance patterns in this species‐rich Bornean forest.

### 4.1 Mechanisms of density dependence

Characterizing the strength of spatial variation in, and specific mechanisms causing, density dependence, is nontrivial. In this study, we found that experimental increases of local seedling density caused increased mortality under normal field conditions, but that application of a fungicide removed these effects (Figure 2a). This interaction suggests that the effects of density on survival are mediated at least in part by soil‐borne fungi, which is consistent with numerous studies (e.g., Augspurger, 1983b; Bagchi et al., 2010). For example, a previous investigation at our study site (focused on physiological underpinnings of interspecific variation in juvenile survival) found that survival of *D*. *aromatica* juveniles actually *increased* with the local‐scale stem density of conspecific juveniles and that this had a stronger effect on survival than any abiotic factors considered (Aiba & Nakashizuka, 2007). Still, because that study was purely observational, the authors could not disentangle the correlated effects of conspecific stem density and distance‐dependent effects of proximity to parent trees. Further, our study assessed much higher densities of *D*. *aromatica*—up to 69 individuals per m^2^ compared to <15 individuals per m^2^ for Aiba and Nakashizuka (2007). This is comparable to reported field densities of 1–183 seeds per m^2^ for mast fruiting dipterocarps (Canon et al., 2020). Thus, the high seedling density we used potentially increased our likelihood of detecting negative relationships between density and performance that would be present during masting fruiting episodes that characterize recruitment in this forest.

Unlike with seedling survival, the weak negative effects of density on seedling growth were unaffected by fungicide, suggesting trends in growth may be driven more by intraspecific competition for limiting resources (e.g., light and nutrients; Clark & Clark, 1984). Evidence is limited for competitive interactions among conspecific seedlings in tropical forests (Paine et al., 2008; Swamy et al., 2010; Terborgh, 2012), although some studies have shown weak intraspecific competition (Svenning et al., 2007). Our results suggest that conspecific seedling density affects both growth and survival to a similar degree (our models explained roughly equivalent variance in both responses), but that density‐dependent effects on growth were more likely driven by factors other than seedling–fungal interactions, whereas density‐dependent mortality was mediated by fungal pathogen infection. Still, disentangling negative density‐dependent growth owing to resource competition versus other factors, such as increased allocation to defense when crowded, is challenging. It is important to note that while *D*. *aromatica* is a common and important species at LHNP, because our experiments used a single species, it is not possible to ascertain with certainty which effects are due to specific traits of this species or the Dipterocarpaceae and which are more general properties of ectomycorrhizal or dominant tree species.

### 4.2 Spatial variation in microbial community composition

Spatial variation in the composition of communities of natural enemies is thought to be an essential component in the maintenance of tropical tree diversity (Connell, 1971; Gilbert, 2002; Janzen, 1970). This spatial variation is thought to be driven predominantly by evolutionarily structured host specificity (Gilbert & Webb, 2007; Liu et al., 2012), as evidenced by differences in seedling recruitment in home versus away sites (Augspurger, 1984; Bagchi et al., 2010; Metz et al., 2010). By contrast, we found that conspecific versus heterospecific recruitment neighborhoods did not affect overall patterns of density‐dependent survival or growth of a dominant tropical tree species. On the surface, the weak interactive effects between neighborhood and density on *D*. *aromatica* survival suggest that the underlying agents of density‐dependent mortality, such as attack by fungal pathogens, may not vary spatially with overstory composition, at least at the scales we examined. However, the effect of fungicide on seedling survival and growth *was* dependent on neighborhood. For example, fungicide reduced survival in heterospecific neighborhoods but had little effect in conspecific neighborhoods (Figure 3), and, when fungal communities were disrupted with fungicide, density‐dependent survival shifted from negative to neutral or weakly positive (Figure 2a). While there was no significant difference in density dependence beneath conspecific and heterospecific trees for *D*. *aromatica*, these results with fungicide point to meaningful spatial variation in the composition of soil fungal communities that has implications for seedling recruitment.

While we cannot confirm how the fungal communities varied in the absence of molecular sequencing data, we expect that spatial variation is likely driven by differential abundance and composition of mycorrhizal fungi and fungal or Oomycete pathogens between conspecific and heterospecific neighborhoods. This is not unexpected, as other studies have shown decreased abundance of host‐specific pathogens (Augspurger & Wilkinson, 2007; Bagchi et al., 2014; Packer & Clay, 2000) and mycorrhizal fungi (Dickie & Reich, 2005; Peay et al., 2012) away from host trees, along with general coturnover of microbial and plant communities (Peay et al., 2013; Russo et al., 2012). Similarly, in our previous shade‐house experimental study at Lambir, EM colonization of dipterocarp seedlings decreased in soils collected from beneath non‐dipterocarp hosts, and application of a Captan fungicide significantly reduced EM colonization on the roots of several different species of dipterocarp seedlings, including *D*. *aromatica* (Segnitz et al., 2020). Assuming Captan fungicide reduced EM colonization similarly in our field manipulation, our experimental findings suggest that the different effects of fungicide in conspecific versus heterospecific neighborhoods could result from differences in the proportion of fungi that are EM and the composition of the EM fungi that were present between neighborhoods. If overall EM inoculum potential was lower in heterospecific neighborhoods, then further reduction in EM abundance as a result of fungicide application could have caused even stronger negative effects on seedlings. In conspecific neighborhoods, any potential increase in mortality from fungicide‐induced reductions in EM abundance may have been offset by the positive effects of reduced pathogen load, causing no net effect on seedling survival. Other studies have also found that net effects of mutualists and pathogens can balance out (Bachelot et al., 2017; Deniau et al., 2018; Liang et al., 2015), but also that individual species can differ significantly in the strength of their positive and negative interactions, particularly depending on mycorrhizal association (Bennett et al., 2017; Klironomos, 2003; Teste et al., 2017). Given the absence of molecular data, there are other alternative explanations of the patterns we find with respect to fungicide and growth neighborhood that we cannot exclude—for example, fungicide could have released more generalist fungal pathogens beneath heterospecific trees. However, we think these alternatives are less consistent with the existing literature. Studies that measure both the effects of microbes on plant growth and use molecular methods to characterize the communities of microbes causing these effects are needed to truly understand the complex interplay of spatially dependent interactions between tree species and different fungal guilds and their consequences at the community level in this forest.

### 4.3 Role of positive feedbacks in local and regional abundance

Variation in the strength of CNDD has been linked to forest‐wide patterns of rarity and abundance (Comita et al., 2010). In the 52‐hectare long‐term forest dynamics plot at Lambir, *D*. *aromatica* is the single most abundant emergent species and dominates on sandy soils that are common in the plot; it accounts for the greatest number of stems >1 cm dbh (~2.5% of all stems) as well as the greatest proportion of basal area (~6.7%) (Itoh et al., 2003; Lee et al., 2002). Where it occurs naturally in Peninsular Malaysia, *D*. *aromatica* is always the dominant emergent species (Kachi et al., 1993).

Consistent with its ability to attain high local density, we found survival and growth of *D*. *aromatica* seedlings were marginally higher in conspecific neighborhoods compared to heterospecific neighborhoods. Similar to cases of tropical monodominance (Connell & Lowman, 1989; McGuire, 2007; Torti et al., 2001), our results suggest that EM root colonization may be facilitated by nearby adult members of the same species. While logistical constraints and seed availability limited us to working with a single EM tree species, other studies have shown that increased distance from parent tree or other conspecific adult EM hosts reduces colonization of EM seedlings in diverse tropical forests (Onguene & Kuyper, 2014). However, the role of EM fungi in dipterocarp seedling growth is species‐dependent, and a series of experiments showed that exclusion from hyphal networks yielded mixed growth responses in different species (Brearley et al., 2017). As *D*. *aromatica* is a regionally abundant species, its lack of apparent CNDD would be consistent with previous work connecting strength of CNDD and PSF to local abundance (Comita et al., 2010; Mangan et al., 2010). Two previous observational studies have included data that suggest the survival of *D*. *aromatica* may actually be increase with abundance of local conspecific seedlings (Aiba & Nakashizuka, 2007) or local conspecific adults (Suzuki et al., 2016).

Recent studies have also shown that there is variation in the prevalence of mycorrhizal symbioses across climate and edaphic conditions (Jo et al., 2019; Steidinger et al., 2019; Weemstra et al., 2020), but also across forests with different evolutionary history (Fukami et al., 2017). If the differences we find for *D*. *aromatica* are consistent across dipterocarps and other EM and AM tree species in the tropics, it is possible that global‐scale variation in tropical forest structure (Banin et al., 2012) may be influenced by the evolutionary history of mycorrhizal symbiosis.

## 5 CONCLUSIONS

While our study cannot definitively conclude that regional abundance of *D*. *aromatica* is reinforced by *strong* positive neighborhood effects, the weight of evidence from this and other studies suggests the view that its recruitment is at least not locally suppressed by the presence of conspecifics. Our results are consistent with this being caused by spatially balanced local positive effects from host‐specific mycorrhizal inoculum and escape from host‐specific pathogens, providing *D*. *aromatica* seedlings many potential recruitment sites. Such effects could facilitate its abundant distribution in this forest, although they cannot explain why this species shows strong local association with particular edaphic habitats. Among other mechanisms, changes in the balance of positive and negative fungal interactions across different soil environments or climates may explain larger scale patterns in dominance for *D*. *aromatica* and other tree species across landscapes or biomes.

## CONFLICT OF INTEREST

The authors declare no conflict of interest.

## AUTHOR CONTRIBUTION

**R. Max Segnitz:** Conceptualization (equal); Data curation (lead); Formal analysis (lead); Investigation (lead); Methodology (equal); Project administration (equal); Visualization (lead); Writing – original draft (lead); Writing – review & editing (equal). **Sabrina E. Russo:** Conceptualization (equal); Funding acquisition (equal); Investigation (equal); Methodology (equal); Project administration (equal); Validation (equal); Writing – review & editing (equal). **Kabir G. Peay:** Conceptualization (equal); Funding acquisition (equal); Investigation (equal); Methodology (equal); Project administration (equal); Resources (equal); Supervision (equal); Validation (equal); Visualization (equal); Writing – review & editing (equal).

## Supporting information

Supplementary Material

## Data Availability

Plant survival, biomass, site information, and treatment data from this study are publicly available through the Dryad data repository on publication (https://doi.org/10.5061/dryad.gxd2547nv).
