# Supplementary material for: Interactions with soil fungi alter density dependence and neighborhood effects in a locally abundant dipterocarp species
Source: Ecol Evol. 2022 Jan 24;12(1):e8478. doi: 10.1002/ece3.8478 (PMC8796921; doi:10.1002/ece3.8478)

SUPPLEMENTAL TABLES

**Table S1:** Analysis of deviance tables for beta-binomial generalized linear mixed effects model on percent survival in the plot network. Table compares model inference between final census data (2018) and the last census before cessation of fungicide treatment (2017). Models include random effect of site.

**Table S2:** Analysis of covariance tables for linear mixed effects models of individual seedling growth responses to experimental treatments. Table compares model inference between final census data (2018) and the last census before cessation of fungicide treatment (2017). Models includes plot-level random effect nested within random effect of site.

**Table S3:** Prediction model for log(biomass) fit by multiple regression. Adjusted R^2^ = 0.92, F_4,162_ = 489.2, p<0.001.

**Table S4:** GLM analyzing treatment effects on probability that seeding mortality was caused by insect stem-clipping.

**Table S5:** Beta-binomial GLMM estimated slopes from simple slopes analysis on the effect of initial cohort density on seedling mortality. Table compares model inference between final census data (2018) and the last census before cessation of fungicide treatment (2017).

**Table S6:** Model selection table for candidate biomass estimation models. Only AIC was used as a selection criterion, though adjusted R^2^ values are presented for comparison. Models 3, 4, and 7 were considered to be equally valid as dAIC among them was <2; however, model 7 was selected as it was the most parsimonious model.

SUPPLEMENTAL FIGURES

F**igure S1:** Interactive effect of neighborhood and fungicide on plot level mortality. Interaction p = 0.13. Trend lines show model prediction estimated from beta-binomial GLMM. Point size indicates the number of seedlings per plot (4-25). P values for individual trends within the interaction are derived from simple slopes analysis.

­

**Fig S2:** Relationships of prediction measurements to log biomass in estimation training dataset.

**Fig S3:** Seedling biomass prediction. Points are partial residuals and blue fit lines indicate the model prediction of the best fit multiple regression. Dashed orange lines show a loess spline fit to the partial residuals, indicating that model fits the data well.


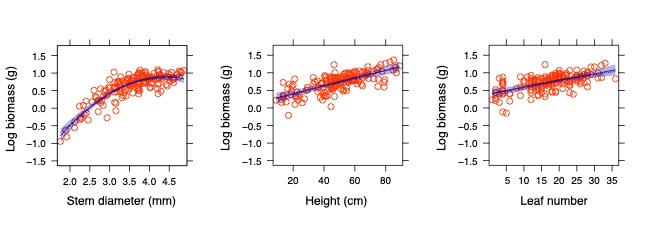


**Fig S4:** Predicted biomass of field seedlings throughout the course of the experiment. Plots indicate relationships between measurements made in the field and the model predicted biomass at five timepoints. Dashed rectangles indicate the ranges growth measurements and of measured dry biomass in the model training data set, reflecting that few predictions fall outside of the training intervals.


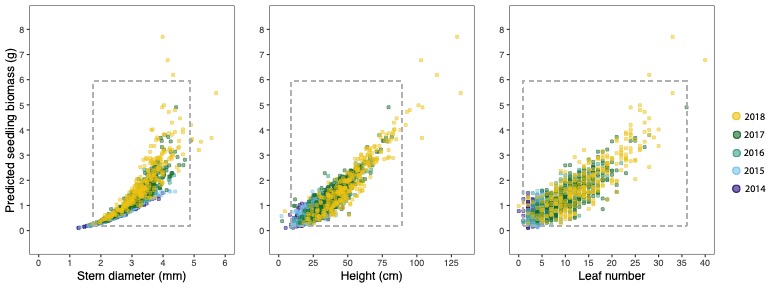

Supplement: Supplementary file 1 — Supplementary Material [file ECE3-12-e8478-s001.docx]
